# Roughness Analysis on Composite Materials (Microfilled, Nanofilled and Silorane) After Different Finishing and Polishing Procedures

**DOI:** 10.2174/1874210601509010357

**Published:** 2015-10-26

**Authors:** Francesco Pettini, Massimo Corsalini, Maria Grazia Savino, Gianluca Stefanachi, Daniela Di Venere, Carmine Pappalettere, Giuseppe Monno, Antonio Boccaccio

**Affiliations:** 1 Dental School - University of Bari, Piazza Giulio Cesare 11 – 70125, Bari, Italy; 2 Department of Mechanics, Mathematics and Management, Politecnico di Bari, Viale Japigia 182, 70126 Bari, Italy

**Keywords:** Composite, finishing, polishing, profilometer, surface roughness.

## Abstract

The finishing and polishing of composite materials affect the restoration lifespan. The market shows a variety of finishing and polishing procedures and the choice among them is conditioned by different factors such as the resulting surface roughness. In the present study, 156 samples were realized with three composite materials, -microfilled, nanofilled and silorane-, and treated with different finishing and polishing procedures. Profilometric analyses were carried out on the samples’ surface, the measured roughness values were submitted to statistical analysis. A complete factorial plan was drawn up and two-way analysis of variance (ANOVA) was carried out to investigate whether the following factors affect the values of roughness: (i) material; (ii) polishing/finishing procedure. Tukey post-hoc test was also conducted to evaluate any statistically significant differences between the material/procedure combinations. The results show that the tested materials do not affect the resulting surface quality but roughness values depend on the finishing/polishing procedure adopted. The procedures that involve: (a) the finishing with medium Sof-Lex discs and (b) the finishing with two tungsten carbide multi-blade milling cutters Q series and UF series are those that allow the lowest values of roughness to be obtained.

## INTRODUCTION

The improvement of the superficial properties of biomaterials has steadily grown in importance in modern industry. Chemical-physical treatments of surface can be utilized to control the events occurring in the interface between materials and the complex environment of biological phases [1]. In particular, in the field of restorative materials, the finishing and polishing phases are compulsory steps in conservative dentistry not only in terms of beauty of the restoration but also in terms of oral health keeping [2, 3]. In the last years, following to the development of nanotechnology, restorative materials have seen a fast and constant evolution in the quantitative and qualitative composition of the filler, the resin mold and in the physical-chemical properties of the composite [4, 5]. The high performances exhibited by composite materials, like microfilled, nanofilled and siloranes, has led to their intensive utilization in Dentistry. The low roughness of the restoration surface represents an essential requirement for the periodontal integrity of teeth, for the marginal integrity of the restoration as well as for its longevity [6-8].

The failure or the absence of appropriate finishing and polishing procedures leads to high values of roughness and hence to unfavorable outcomes such as:

- dental plaque accumulation;

- less endurance to wear;

- gum irritation;

- poor esthetic quality;

- color alteration.

Relationships between polishing/finishing protocols, thermal treatments and surface roughness were investigated in the literature [9-1]. It appears that the roughness of restoration surface is mainly affected by polishing and/or finishing technique and operator’s manual skills. Standardized methods of polishing a surface independently from individual operator’s skills have been proposed [12, 13].

The aim of the present study is to assess how the roughness of a restoration surface changes for different finishing and polishing procedures and for different materials it is made from. 

## MATERIALS AND METHODS

### Materials and Finishing/Polishing Procedures Tested in the Study

The roughness parameters have been investigated for the following materials:

- ***ESTHETX HD:*** Photopolymerizable micro-hybrid composite, radiopaque, utilized in the restoration of the anterior and posterior zones.
- ***CERAMX MONO:*** Photopolymerizable nano-hybrid composite, used in direct and indirect restorations of anterior and posterior zones, for splinting and tooth stub restoration.
- ***FILTEK SILORANE:*** Photopolymerizable silorane composite, used in the restoration of the posterior zone.

A large variety of finishing and polishing tools are currently available on the market. Some of these have been investigated in the present study.

- ***Tungsten Carbide Multi-blade Milling Cutters:*** These devices are classified according to the number of blades (from 8 to 30). The cutting tool includes an entry and exit angle and to correctly work, must advance in the counter-rotation sense;
- ***Diamond Milling Cutters:*** These cutters are produced in different shapes and dimensions and with different types of grain (the dimensions of which range in the interval 8 - 50 μm). They are usually utilized in sequence; the cutter with the higher granulometry (50 - 30 μm) is used to rough-hew and mold the restoration. The final finishing is then performed with a lower granulometry (8 - 15 μm) cutter. The shape of the cutter changes according to the part to finish; flame-shaped milling cutters are usually utilized for vestibular zones, olive-shaped milling cutters for the palatal ones.
- ***Abrasive Wheels:*** These are flexible, decreasing granulometry wheels for finishing and polishing, with different gauges and therefore, different rigidity. Each granulometry is associated with a different color. They can be used singularly or in combination with diamond or multi-blade milling cutters. The most common discs are made of a paper frame sprinkled with abrasive (oxide alloy) of different grain. The discs have a small eyelet to be click-inserted to the spindle mounted on the hand-piece. The finishing and polishing will be performed in three or four operative steps. The wheels used in the present study were Sof-lex (3M, St. Paul, MN, USA). The system includes wheels with four types of oxide alloy grain, from rough to superfine.
- ***Silicon Rubber Tips:*** These are flexible rubber tips composed of a silicon mold to which carbide silicon, oxide alloy or diamond abrasive particles are added. These devices are strictly used for the final finishing and polishing; they are available on the market in different colors, shapes and grain dimensions (15 - 40 μm). They are mounted on the micromotor and used under large amounts of water spray. In addition to the different grain tips used in the multi-step procedure, the one-step silicone rubber tip was recently introduced on the market. Once the finishing is terminated, the operator utilizes the one-step silicone rubber to obtain a mirror polishing.
- ***Polishing Pastes:*** These are diamond or oxide alloy pastes with different grains. They have to be dry-applied on the composite one or more times with discs, plastic tips or goat-hair brushes.

The main features of the adopted procedures, the producer and batch number (of the items utilized in the experiments) are listed in Table **1**. 

### Preparation of Samples

For each tested material 52 samples were realized, using thermoplastic resin molds. The molds were built by using the following items/devices:

- ***Nails:*** The nail head (with a diameter of 8 mm and a thickness of 2 mm) was used as a model for the molds;
- ***Extrastrong and Soft Chalk*** (Elite Dental Stones, Zhermack Spa, Badia Polesine (RO), Italy);
- ***Thermoplastic Resin Discs*** with a 125.0 mm diameter and a 3.0 mm thickness (Imprelon, Scheu Dental GmbH, Iserlohn, Germany)
- ***Infrared Heater:*** Biostar (Scheu Dental GmbH, Iserlohn, Germany).

The following procedure was adopted.

**Fig. (1):**
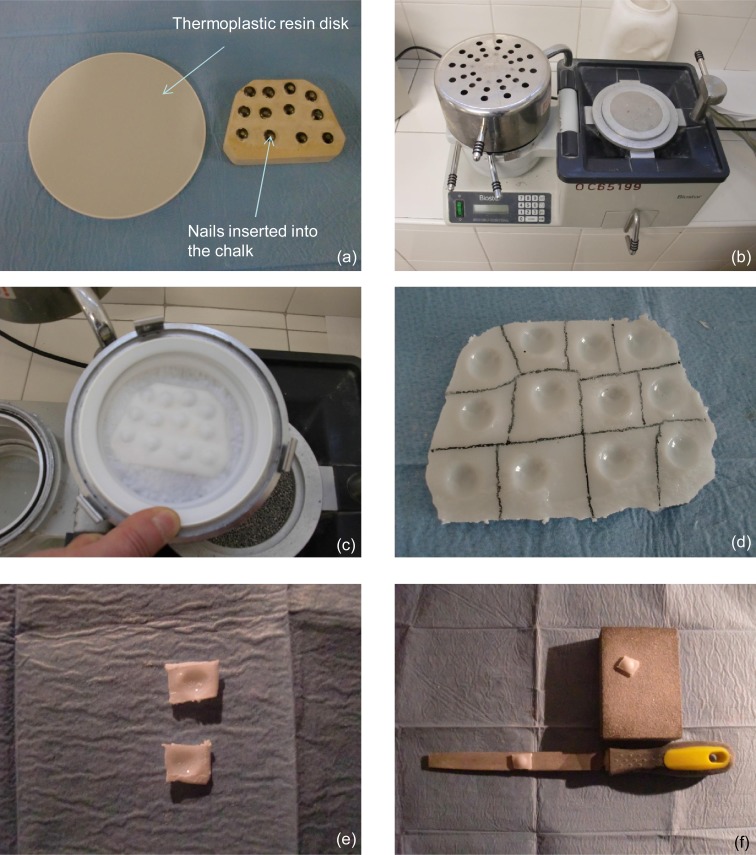
Principal phases during the preparation of the samples. (a) A prismatic model was first built with impression chalk within which different nails were inserted. Thermoplastc resin disks were heated with the Biostar System (b) and inserted into the model at a pressure of 4.1 atm so as to reproduce the dimension of the nail head (c). After being shaped and once the cooling was completed, the discs were removed (d). The discs were finally cut with a milling cutter in many different squares (d-e) the surface of which was then polished (f).

1. With the impression chalk, a prism was realized in which the nails were inserted (Fig. **1(a)**);
2. Thermoplastic disks were heated with the Biostar system at the temperature of 220° C for 90 seconds thus losing their initial consistency (Fig. **1(b)**);
3. The discs were inserted into the model at a pressure of 4.1 atm so as to reproduce the shape of the nail head (Fig. **1(c)**);
4. After being shaped and once the cooling was completed, the discs were finally removed (Fig. **1(d)**);
5. The discs were finally cut with a milling cutter in many different squares (Fig. **1(d-e)**) the surface of which was then polished (Fig. **1(f)**).

**Fig. (2):**
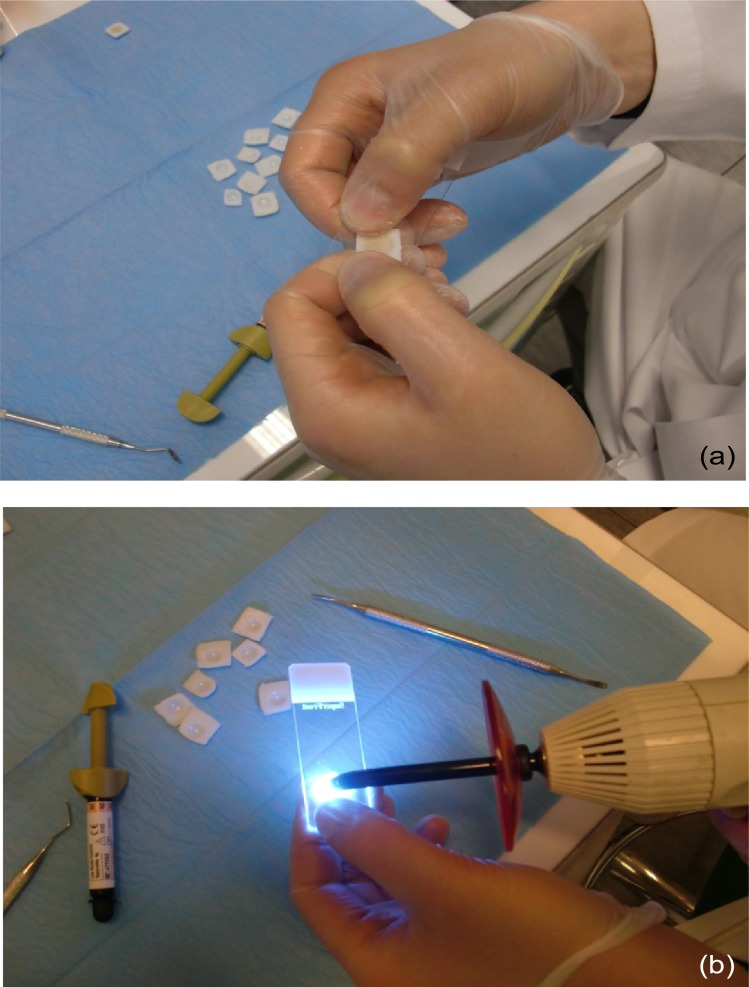
(a) The composite materials were put in the molds with a putty knife and covered with a Mylar strip and a microscope slide. (b) The samples, together with the strip and the slide, were radiated for 40 seconds by a halogen lamp.

The composite materials were hence put in the molds with a putty knife and covered with a Mylar strip and a microscope slide (Fig. **2(a)**). The strip was utilized so as to avoid the formation of an oxygen inhibition layer, whereas through the slide a load of about 20 N was applied on the composite surface thus enabling the consolidation and the discharge of material’s excess.

The samples, together with the strip and the slide, were radiated for 40 seconds (according to the Manufacturer’s prescriptions), by a halogen lamp (Degulux soft-star, Degussa-Hülls, Hanau, Germany) placed perpendicularly with respect to the sample surface and working at an intensity greater than 600 mW/cm^2^ (Fig. **2(b)**). After the polymerization was completed, the samples were kept for 24 hours at the temperature of 37°C and 100 % of humidity so as to complete the process and then dried.

### Finishing and Polishing of the Samples

After examining the samples to check any possible inner voids, the samples were finished and polished by adopting the following procedures:

- ***Procedure A***: (control group): A control group including 4 samples was collected for each of the tested composite materials. All the samples of the three control groups were neither finished nor polished after polymerization.
- ***Procedure B***: 16 samples for each composite were treated with this procedure. The finishing procedure was carried out for thirty seconds with medium Sof-Lex (3M, St. Paul, MN, USA) discs. The discs, according to the Producer’s guidelines, were mounted on the micromotor together with the spindle. The discs were then used with uniform and regular movements from the restoration towards the margin (and not vice-versa) avoiding forward-backward movements on the composite/mold margin which could have resulted in the formation of a white line and hence of a discontinuous region. The whole procedure was operated in the absence of water. At the end of the finishing process, the samples were washed and dried with an airflow. Twelve samples (four for each composite) were not polished (Group B1). The remaining samples (Group B2) were polished as follows:

- 12 samples (4 for each composite) were polished for 30 seconds with fine and superfine Sof-lex discs always inserted to the spindle and mounted on the hand-piece (Group B2I).

- 12 samples (4 for each composite) were polished for 30 seconds with a silicone rubber tip. The rubber tip was used with slightly circular movements at a speed of 6000 rpm, under an adequate refrigeration obtained with a 50 ml/min water flow. A special care was taken in minimizing the pressure exerted with the disc on the polished surface (Group B2II);

- 12 samples (4 for each composite) were polished with the following procedure:

- Superficial degreasing with 95° alcohol;

- Application of Experience seal coat (DEI Italia S.r.l., Mercallo (VA), Italy) on the surface with circular movements with a microbrush gently rubbing it until the solvent evaporated;

- Removal, after 3 minutes, of the surplus with a new microbrush and photopolymerization for 90 seconds with a halogen lamp (Degulux soft-star).

- Dry-application of Experience polish paste (DEI Italia S.r.l.) with soft goat-hair wheel until the complete removal of any residual seal coat fast;

- Dry-polishing with cotton mop and Experience Polish Paste (Group B2III).

***Procedure C***: 16 samples for each composite were treated with this procedure. The samples were finished with two flame-shaped diamond milling cutters: firstly, a 50 μm (red ring) rough-grain, then a 30 μm (yellow ring) extrafine grain milling cutter was utilized. Each milling cutter operated for thirty seconds. The cutters were mounted on the turbine and used under abundant irrigation. After the finishing, the samples were washed and dried with an airflow. Twelve samples (four for each composite) were not polished (Group C1). The remaining samples (Group C2) were classified and polished according to the scheme utilized for Procedure B and summarized in Table **2**. For instance, Group C2I includes 12 samples (4 for each composite) polished for 30 seconds with fine and extrafine-grain Sof-lex discs inserted into the spindle and mounted on the hand-piece. Analogously, Group C2II includes 12 samples polished for 30 seconds with a silicone rubber tip, while Group C2III includes 12 samples submitted to the same treatment as that utilized for samples of Group B2III (Table **2**).

***Procedure D***: 16 samples for each composite were treated with this procedure. The samples were finished with two tungsten carbide multi-blade milling cutters Q series (yellow-blue) and UF series (white) for 30 seconds for each milling cutter. They were mounted on the turbine and used under plentiful irrigation. After the finishing was completed, the samples were washed and dried with airflow. Twelve samples (four samples for each composite) were not polished (Group D1). The remaining samples (Group D2) were polished according to the scheme shown in Table **2**.

In order to reduce the variability of the preparation procedures, finishing and polishing were performed by the same operator. At the end of these phases the samples were washed with distilled water and left drying for 24 hours before the roughness analysis.

### Roughness Analysis

**Fig. (3):**
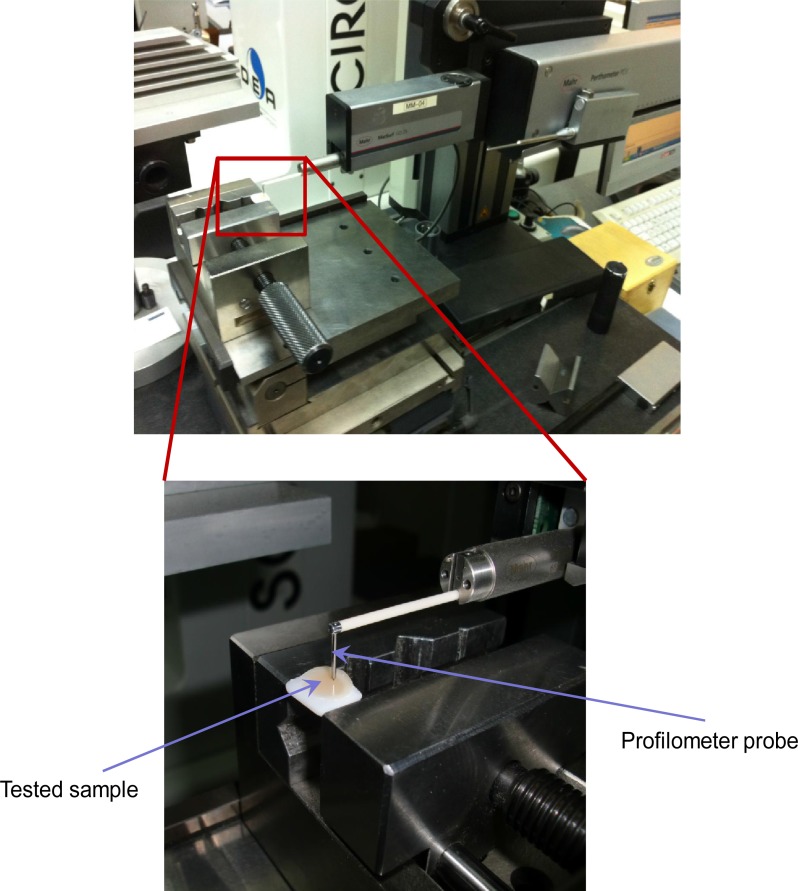
The profilometer Mahr MarSurfGD25 while measuring the roughness of a sample. The profilometer probe comes in contact with the surface detecting the profile of the scanned path.

Any surface, even if worked with great accuracy, when observed with a microscope will reveal roughness due to grooves and crests determining local distancing, more or less extended, of the real surface from the one conceived in the design. Roughness (or absolute roughness) is a property of the object surface, it consists of geometric micro-imperfections either originally included in the material of 

which the surface is made or due to the manufacturing process. These imperfections usually take the form of grooves or scratches varying in shape, depth and direction. In order to assess the roughness of a surface, a profilometer is used. The measuring procedure is performed in the recording of the profile of the surface along a specific measuring path (or scanning path). This profile is then analyzed defining a numerical parameter which is the roughness value. A crucial phase of the roughness measurement process is the filtering procedure which allows the assessment of the sole quality of the surface to be separated from the effects that the geometrical errors have on the measured profile. In the present study, after the finishing and polishing were performed, the superficial roughness of the samples was measured with the profilometer Mahr MarSurfGD25 (Mahr Inc., Gottingen, Germany) (Fig. **3**). The measuring conditions, according to ISO (International Standards Organization) 4287 Standard were:

- Value of the profilometric resolution: 0.01 μm
- Transverse length (TL): 5.6 mm (n=5)
- Sample length (SL): 2.5 μm
- Vertical band width: ±250 μm
- Scanning Rate Vt: 0.50 mm/s
- Number of acquired points: 11200

The assessed parameter was Ra, i.e. the average arithmetic roughness. It is the most common parameter and represents the average absolute value of the deviations of the detected surface from the “average value” of the profile (technical surface) (ISO 4287). Also in this case, all the roughness measurements were carried out by the same operator. 

### Statistical Analysis

**Fig. (4):**
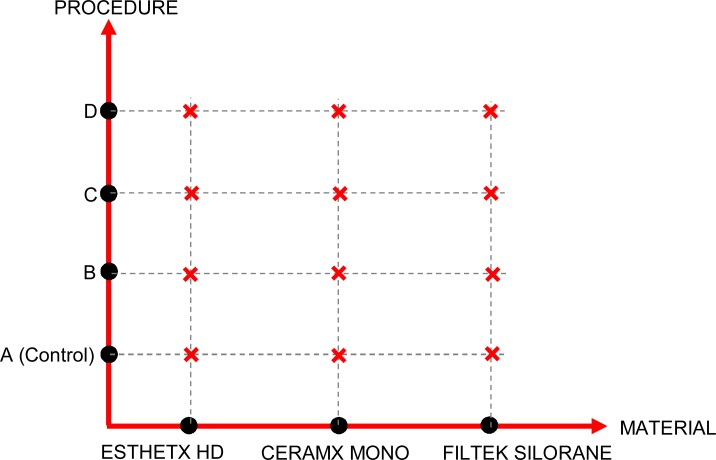
Complete factorial plan designed to identify the factors that have a statistically significant influence on the roughness surface.

The statistical analysis was aimed at identifying, by two-way analysis of variance (ANOVA), the factors that affect the quality of the restoration surface. A complete factorial plan was drawn up (Fig. **4**), assessing two factors: (i) material; (ii) procedure. The null hypothesis *H_o_* was that factors (i) and (ii) do not affect the roughness values. *H_o_* was assumed to hold true for *p-values *≥ 0.05 (95 % confidence interval). For factor (i) three levels were fixed: ESTHETX HD, CERAMX MONO and FILTEK SILORANE; for factor (ii) four levels: procedure A, B, C and D. A total of 156 roughness values were submitted to statistical analysis: 12 values for Procedure A 4 (replications) × 3 (number of tested composite materials); 48 values for Procedure B 4 (replications) × 4 (number of sub-groups, i.e. B1, B2I, B2II, B2III) × 3 (number of tested composite materials); 48 values for Procedure C 4 (replications) × 4 (number of sub-groups, i.e. C1, C2I, C2II, C2III) × 3 (number of tested composite materials); 48 values for Procedure D 4 (replications) × 4 (number of sub-groups, i.e. D1, D2I, D2II, D2III) × 3 (number of tested composite materials). 

A non-parametric test (analysis of variance on ranks with Tukey post-hoc test) was utilized to compare the roughness values obtained with the different procedures and materials. 

## RESULTS

Two-way ANOVA revealed that the factor (ii) procedure, p-value = 0.000 certainly does affect the roughness values while roughness seems to be rather insensitive to the factor (i) material, p-value = 0.109. For each of the investigated material/procedure combinations, the median value, the first quartile, third quartile, minimum and maximum values are indicated in the boxplot of Fig. (**5**). In the Tables **3**, **4**, **5** and **6** we report the mean values with the standard deviation of Ra evaluated in the procedures A, B, C and D, respectively, for all the investigated materials. The mean roughness values measured for different levels of factors (i) and (ii) are shown in the main effects plot (Fig. **6**). The highest values of Ra were found for samples treated with procedure A, i.e. for samples neither finished nor polished after polymerization. Tukey post hoc test showed that there were statistically significant differences between the roughness measured on samples treated with procedure A and that measured on samples submitted to procedures B and D. No statistically significant differences could be seen between the Ra values of procedure A and those of procedure C (Table **7**). However, averagely, the roughness values found for samples submitted to procedure C are smaller than those measured in the case of the procedure A (Fig. **6, **Table **7**). Furthermore, no statistically significant differences have been found between the roughness values obtained for different materials. The average values of roughness measured on samples submitted to polishing (in addition to finishing) are approximately 10-20 % smaller than those measured on samples solely finished (Fig. **7**). 

## DISCUSSION

In the present study, different composite materials were submitted to polishing and finishing procedures. Roughness measurements were carried out by means of a profilometer and the resulting roughness values were submitted to statistical analysis. A two-way ANOVA was utilized to analyze whether the surface roughness depends on the material it is made from and the procedure with which it has been treated. 

In order to understand the values measured in the study it must be taken into consideration the fact that the superficial micromorphology obtained after finishing and polishing is affected by some factors [14-16].

- Polymerization-related factors: the main controversy related to the finishing and polishing of composites is about the time to begin these procedures. Whereas some authors claim that finishing and polishing have to be performed after the removal of the mold or in the following five minutes, other authors advise for a 24 hour-delay as not to damage the margin of the restoration. Furthermore, according to these authors, immediate finishing and polishing could result in a flux in composites due to thermal perturbations [17]. After polymerization, composites are subjected to hygroscopic expansion which reduces microinfiltrations [18]. Performing the finishing and polishing at a later stage would nonetheless compromise the marginal sealing obtained with the hygroscopic expansion of the composite and adhesive system, engendering an increase in microinfiltrations. Instant procedures instead would compromise the initial marginal keeping, still the hygroscopic expansion could contain the situation [19].
- Finishing and polishing procedures-related factors such as: abrasive hardness, geometry, flexibility and application speed of the polishing/finishing tools [20-22]. Consistently with this, in the present study statistically significant differences were found between the values of Ra obtained on samples submitted to different finishing/polishing tools and/or procedures.
- Operator-related factors: studies in literature are conflicting because according to some authors the operator’s age and experience do not seem to affect the polishing quality and consequently the superficial characteristics. For other authors the operator’s experience and expertise can also affect the final superficial roughness degree [12, 13].

**Fig. (5):**
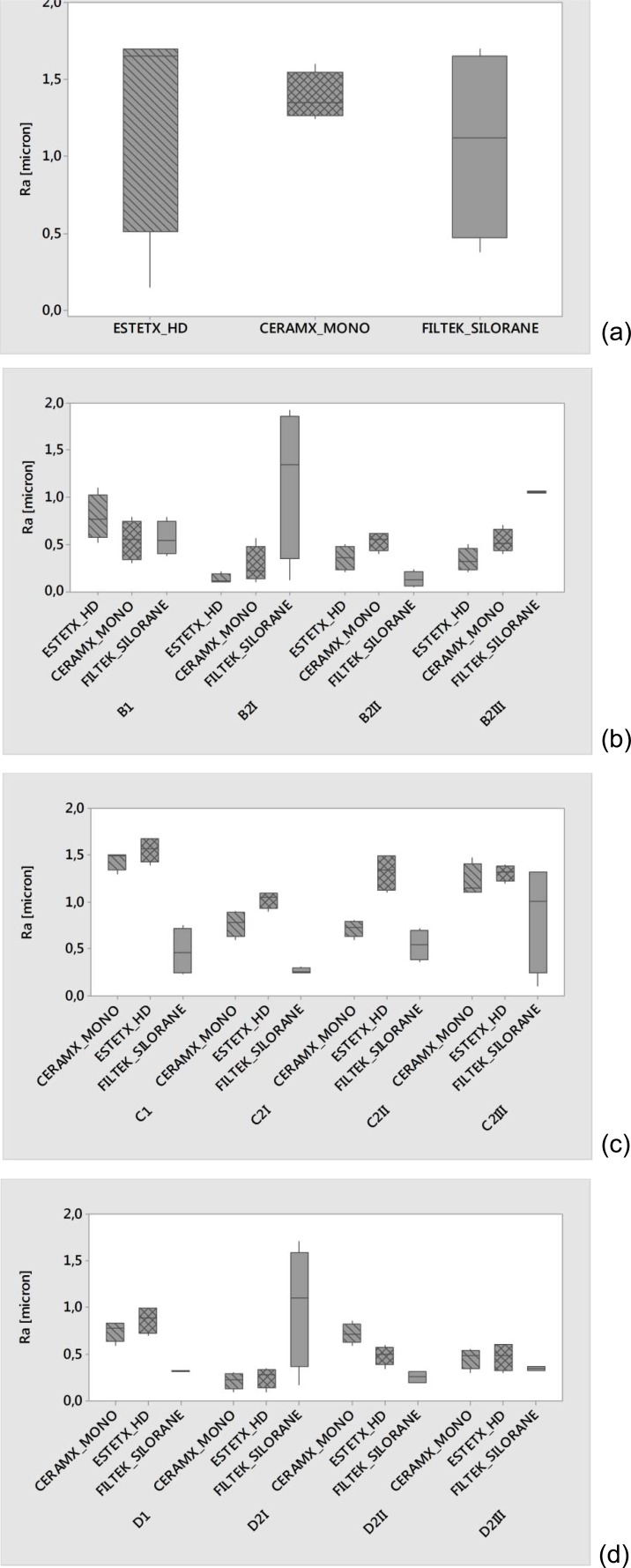
Boxplot of roughness values determined for the different groups of tested samples: (a) procedure A; (b) procedure B; (c) procedure C; (d) procedure D.

**Fig. (6):**
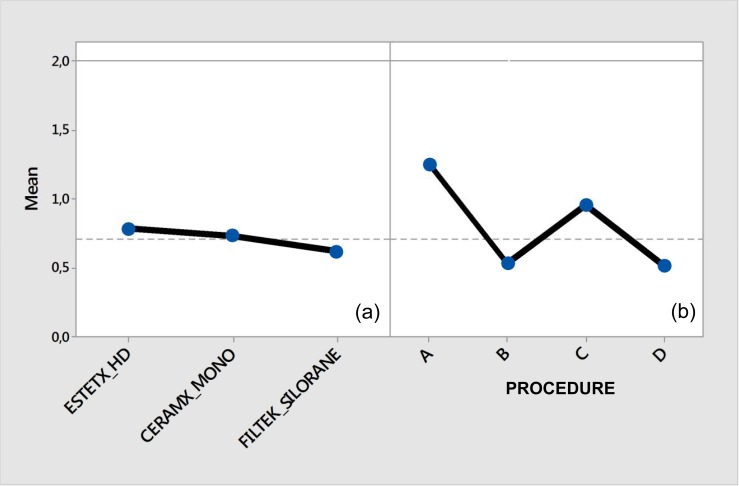
Main effects plot reporting the values of roughness averaged over the factors (i) materials and (ii) procedure. The horizontal line reported in the two diagrams represents the average roughness value measured in all the experiments conducted. For example, the value of roughness reported in the diagram (a) for the material CERAMX MONO, represents the average roughness value measured on all the samples made of this material. Similarly, the roughness value reported in the diagram (b) for the procedure B represents the value of roughness averaged over all the measurements carried out on samples treated with this procedure.

**Fig. (7):**
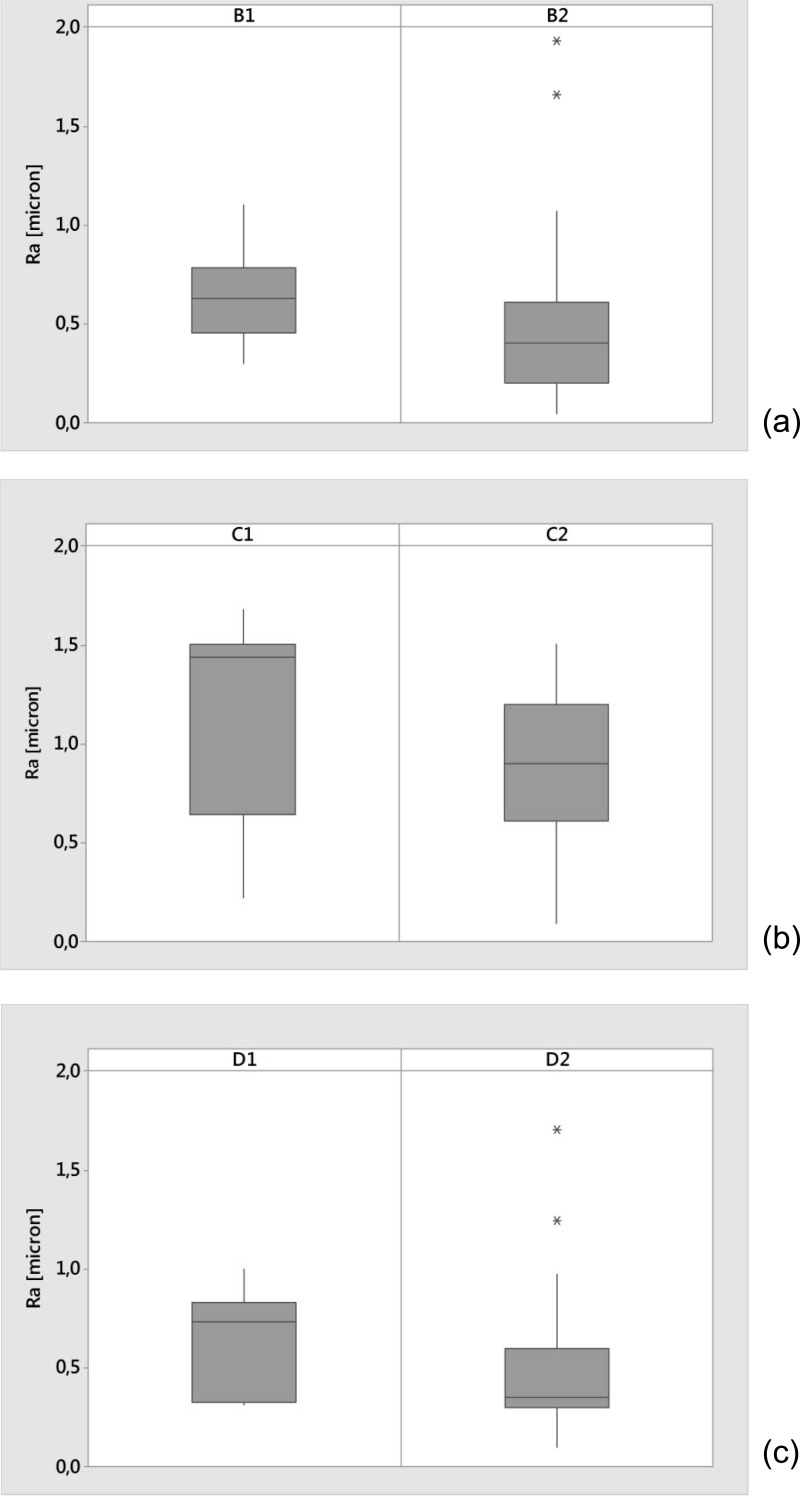
Boxplot of roughness values measured for the samples treated with procedure B (a), C (b) and D (c) in the case of polishing included (B2, C2, D2) or excluded (B1, C1, D1). The boxplot that refers to group B2 includes the roughness values obtained for the sub-groups: B2I, B2II and B2III. Analogously, groups C2 and D2 include the sub-groups C2I, C2II, C2III and D2I, D2II, D2III, respectively.

The roughness analysis carried out in the present study showed that the finishing and polishing procedures have certainly beneficial effects on the quality of the restoration surface. The average value of Ra measured for samples treated with procedure A (control group) is about twice as large as that measured for procedure B and D. The statistical analysis showed no statistically significant differences between the samples treated with procedure A and those treated with procedure C, however, the values of Ra measured for the first samples were averagely 20 % higher than those measured for the second ones (Fig. **6(b)**). ANOVA showed that the tested materials do not affect the resulting surface quality but roughness values depend on the finishing/polishing procedure adopted. It can be seen, in fact, that the average values of Ra obtained for the three tested materials are practically overlapping (Fig. **6(a)**) while change significantly for different finishing/polishing procedures (Fig. **6(b)**). The procedure assuring the best results was procedure B and procedure D (Fig. **5** and **6**). In all the procedures tested in the study, the polishing operation allows values of roughness averagely smaller than those of samples solely finished, to be obtained (Fig. **7**).

**Fig. (8):**
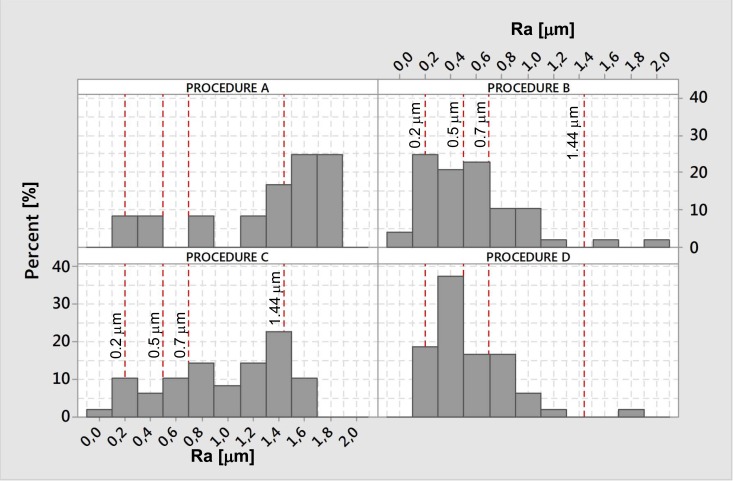
Histograms showing, for each procedure investigated, the percentage of samples the roughness of which falls within given intervals. For example, with reference to procedure A, about 8 % of samples presents a roughness within the interval 0.1 - 0.3 mm; similarly, with reference to procedure D, about 37 % of the samples present a roughness that falls within the interval 0.3 - 0.5 mm. The dashed red lines represent threshold roughness values reported in the literature above which a simultaneous increase in the plaque and in the risk of caries and periodontal inflammation is associated.

Assessing these values is of great importance since a missing finishing and polishing causes a series of negative occurrences, among which the adhesion of bacterial plaque to the composite [2] and the consequent onset of secondary caries. Many studies show a strong correlation between the material roughness and the initial adhesion of bacterial plaque. The minimum roughness value still remains unclear. Some authors claim it to be 0.7-1.44 μm, others 0.25-0.50 μm or 0.2 μm [23, 24]. Considering that any reduction in bacterial accumulation is predicted under these thresholds, any increase in the superficial roughness above these values is associated with a simultaneous increase in the plaque and in the risk of caries and periodontal inflammation [2]. Interestingly, a large number of samples with roughness exceeding 1.44 μm has been found only in the case procedure A (Fig. **8**). 50 % (note: this value can be easily determined by summing up the percentages related to the histograms with Ra > 1.44 μm) of samples treated with procedure A, in fact, present Ra values greater than 1.44 μm (Fig. **8**). The percentage of samples with Ra > 1.44 μm is about 32 %, 2 % and 1 %, in the case of procedures C, B and D, respectively. A wide incidence of samples with Ra < 0.7 μm, can be found only for procedures B and D (Fig. **8**). 

The proposed study presents some limitations. First of all, all the polishing/finishing procedures were carried out by the same expert operator. In spite of the care taken in carrying out all the operations according to the prescribed protocol, it is reasonable to hypothesize that the quality of the work done may depend on “human” factors such as the level of attention, wrist trembling, that cannot be controlled. Secondly, the roughness analysis was carried out by using a profilometer that detects the shape of the surface along specific paths. The utilization of new technologies [25] based on optical methods would enable to measure the roughness not only on paths but on entire areas thus allowing to significantly increase the number of sampled points. 

## CONCLUSION

1) Different composite materials were submitted to polishing and finishing procedures. Roughness measurements were carried out by means of a profilometer and the resulting roughness values were submitted to statistical analysis.

2) ANOVA showed that the tested materials do not affect the resulting surface quality but roughness values depend on the finishing/polishing procedure adopted.

3) The roughness analysis showed that the finishing and polishing procedures have certainly beneficial effects on the quality of the restoration surface. The average value of Ra measured for samples neither finished nor polished is about twice as large as that measured for procedure B (finishing with medium Sof-Lex discs) and D (finishing with two tungsten carbide multi-blade milling cutters Q series and UF series) and 20 % higher than that obtained with procedure C (finishing with two flame-shaped diamond milling cutters).

4) The procedures that involve: (i) the finishing with medium Sof-Lex discs and (ii) the finishing with two tungsten carbide multi-blade milling cutters Q series and UF series are those that allow the lowest values of roughness to be obtained.

5) The polishing operation allows values of roughness averagely smaller than those of samples solely finished, to be obtained.

## Figures and Tables

**Table 1.** Schematic of the principal features of the polishing/finishing procedures adopted in the experiments.

Finishing Procedures	Composition	Producer	Batch N.
Sof-lex middle-grain abrasive discs	Oxide alloy	3M ESPE, St. Paul, MN, USA	127683
Tungsten carbide multi-blade milling cutters: Q series + UF series	Tungsten carbide	KOMET ITALIA, Milan, ITALY	860168 871818
Diamond milling cutters Red ring 50 μm Yellow ring 30 μm	Diamond	KOMET ITALIA, Milan, ITALY	884220 852330
Polishing Procedures	Composition	Producer	Batch N.
Sof-lex, fine and extrafine grain abrasive discs (multi-step procedure)	Oxide alloy	3M ESPE, St. Paul, MN, USA	127683
Silicone rubber tip (one-step procedure)	Ultrafine diamond grain rubber tip with flexible silicone binder	KOMET ITALIA, Milan, ITALY	867287
Experience seal coat, Experience polish paste (+ goat-hair brush and cotton mop (multi-step)	Experience seal coat: resine acrilate e metilmetacrilate Experience polish paste: oxide alloy, water, glycerine and sweetener.	DEI Italia, Mercallo, VA, ITALY	5105695 2011003569

**Table 2.** Groups tested in the study.

	No Polishing	Polishing with fine and superfine Sof-lex discs	Polishing with silicone rubber tip	Application of Experience seal coat, Experience polish paste (+ goat-hair brush and cotton mop (multi-step))
Procedure B Samples finished with medium Sof-Lex discs	B1	B2I	B2II	B2III
Procedure C Samples finished with two flame-shaped diamond milling cutters	C1	C2I	C2II	C2III
Procedure D Samples finished with two tungsten carbide multi-blade milling cutters Q series and UF series.	D1	D2I	D2II	D2III
Procedure A Samples neither finished nor polished after polymerization	Control Group			

**Table 3.** Average roughness Ra and standard deviation measured for procedure A (control group, samples neither finished nor polished).

	Procedure	Ra [μm]	Std dev [μm]
ESTHET X HD	A	1.288	0.758
CERAM-X MONO	A	1.389	0.153
FILTEK SILORANE	A	1.081	0.622

**Table 4.** Average roughness Ra and standard deviation measured for procedure B (samples finished with middle grain abrasive discs and successively polished).

	Procedure	Ra [μm]	Std dev [μm]
ESTHET X HD	B1	0.787	0.240
CERAM-X MONO	B1	0.543	0.211
FILTEK SILORANE	B1	0.558	0.1812
			
ESTHET X HD	B2I	0.129	0.048
CERAM-X MONO	B2I	0.272	0.199
FILTEK SILORANE	B2I	1.183	0.802
			
ESTHET X HD	B2II	0.3545	0.127
CERAM-X MONO	B2II	0.528	0.099
FILTEK SILORANE	B2II	0.126	0.078
			
ESTHET X HD	B2III	0.328	0.125
CERAM-X MONO	B2III	0.529	0.125
FILTEK SILORANE	B2III	1.055	0.011

**Table 5.** Average roughness Ra and standard deviation measured for procedure C (samples finished with diamond milling cutters and successively polished).

	Procedure	Ra [μm]	Std dev [μm]
ESTHET X HD	C1	1.556	0.130
CERAM-X MONO	C1	1.444	0.096
FILTEK SILORANE	C1	0.469	0.249
			
ESTHET X HD	C2I	1.023	0.091
CERAM-X MONO	C2I	0.764	0.139
FILTEK SILORANE	C2I	0.257	0.029
			
ESTHET X HD	C2II	1.318	0.198
CERAM-X MONO	C2II	0.712	0.085
FILTEK SILORANE	C2II	0.535	0.166
			
ESTHET X HD	C2III	1.311	0.084
CERAM-X MONO	C2III	1.218	0.176
FILTEK SILORANE	C2III	0.853	0.585

**Table 6.** Average roughness Ra and standard deviation measured for procedure D (samples finished with tungsten carbide multi-blade milling cutters and successively polished).

	Procedure	Ra [μm]	Std dev [μm]
ESTHET X HD	D1	0.868	0.071
CERAM-X MONO	D1	0.751	0.105
FILTEK SILORANE	D1	0.319	0.008
			
ESTHET X HD	D2I	0.252	0.108
CERAM-X MONO	D2I	0.212	0.085
FILTEK SILORANE	D2I	1.022	0.644
			
ESTHET X HD	D2II	0.485	0.103
CERAM-X MONO	D2II	0.721	1.103
FILTEK SILORANE	D2II	0.257	0.060
			
ESTHET X HD	D2III	0.475	0.151
CERAM-X MONO	D2III	0.455	0.109
FILTEK SILORANE	D2III	0.342	0.021

**Table 7.** Average values (computed over the four procedures) and standard deviation of roughness Ra measured in the experi-mental tests. Statistical comparisons (Tukey post-hoc test, Control Group A) are shown in the last column.

	A [mm]	B [mm]	C [mm]	D [mm]	Comparison vs A
mean	1.253	0.533	0.955	0.513	B, D
Std dev	0.535	0.401	0.449	0.316	
